# N-3 long-chain polyunsaturated fatty acids and risk of all-cause mortality among general populations: a meta-analysis

**DOI:** 10.1038/srep28165

**Published:** 2016-06-16

**Authors:** Guo-Chong Chen, Jing Yang, Manfred Eggersdorfer, Weiguo Zhang, Li-Qiang Qin

**Affiliations:** 1 Department of Nutrition and Food Hygiene, School of Public Health, Soochow University, Suzhou, China; 2 Department of Nutrition, the First Affiliated Hospital of Soochow University, Suzhou, China; 3 DSM Nutrition Products, Human Nutrition and Health, Kaiseraugst, Switzerland; 4 DSM Nutrition Products, Human Nutrition and Health, Beijing, China

## Abstract

Prospective observational studies have shown inconsistent associations of dietary or circulating n-3 long-chain polyunsaturated fatty acids (LCPUFA) with risk of all-cause mortality. A meta-analysis was performed to evaluate the associations. Potentially eligible studies were identified by searching PubMed and EMBASE databases. The summary relative risks (RRs) with 95% confidence intervals (CIs) were calculated using the random-effects model. Eleven prospective studies involving 371 965 participants from general populations and 31 185 death events were included. The summary RR of all-cause mortality for high-versus-low n-3 LCPUFA intake was 0.91 (95% CI: 0.84–0.98). The summary RR for eicosapentaenoic acid (EPA) and docosahexaenoic acid (DHA) intake was 0.83 (95% CI: 0.75–0.92) and 0.81 (95% CI: 0.74–0.95), respectively. In the dose-response analysis, each 0.3 g/d increment in n-3 LCPUFA intake was associated with 6% lower risk of all-cause mortality (RR = 0.94, 95% CI: 0.89–0.99); and each 1% increment in the proportions of circulating EPA and DHA in total fatty acids in blood was associated with 20% (RR = 0.80, 95% CI: 0.65–0.98) and 21% (RR = 0.79, 95% CI: 0.63–0.99) decreased risk of all-cause mortality, respectively. Moderate to high heterogeneity was observed across our anlayses. Our findings suggest that both dietary and circulating LCPUFA are inversely associated with all-cause mortality.

The potential benefits of marine derived n-3 long-chain polyunsaturated fatty acids (LCPUFA) (e.g., eicosapentaenoic acid [EPA] and docosahexaenoic acid [DHA]) have received great attention for over 40 years^1^. Prospective observational studies have shown consistent associations between intake of fish, a major dietary source of n-3 LCPUFA, and reduced risk of cardiovascular disease (CVD), in particular of coronary heart disease (CHD) mortality^2^. Experimental and human intervention studies have demonstrated that n-3 LCPUFA have various physiologic benefits, such as improvement of endothelial function^3^ and reductions of heart rate, blood pressure, and inflammation^4^,^5^. Some early randomized controlled trials (RCT) find significant reductions in cardiac death by n-3 LCPUFA supplementation^6^. The encouraging evidence led to the American Heart Association to recommendation to increase oily fish intake or n-3 LCPUFA supplementation for primary and secondary prevention of CHD^7^. Such a recommendation, however, has been questioned by several recent human intervention trials^8^,^9^ and meta-analyses^10^,^11^ of available RCTs. Potential explanations for the differences between observational and clinical studies, and between early and recent trials are addressed in greater depth below.

Circulating concentrations of n-3 LCPUFA represent a good indicator of dietary intakes and endogenous metabolism^12^. There have been a number of prospective observational studies that investigate the relationships between dietary or circulating n-3 LCPUFA and all-cause mortality, and the results have been inconsistent^13^,^14^,^15^,^16^,^17^,^18^,^19^,^20^,^21^,^22^. In an attempt to quantitatively summarize the evidence, a systematic review and meta-analysis was carried out in the present study.

## Results

### Study selection and characteristics

**Figure 1:**
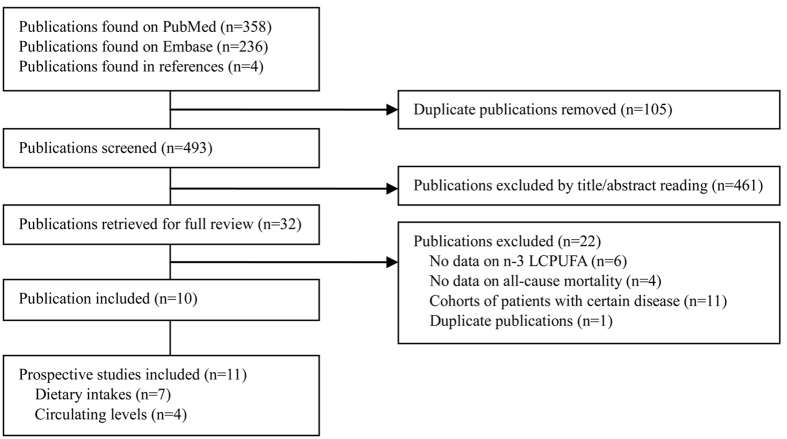
Literature search for the meta-analysis. LCPUFA, long-chain polyunsaturated fatty acids.

A flow chart of study selection is reported in Fig. 1. Briefly, a total of 493 independent citations were identified after duplicate exclusion, of which 32 were retrieved for full-text review. Ten publications were excluded because the exposure or outcome was not relevant to the topic we studied; 11 reports were excluded because they investigated dietary/circulating n-3 LCPUFA among patients with certain diseases, such as type 2 diabetes^23^,^24^, CHD^25^,^26^,^27^,^28^, heart failure^29^,^30^, and kidney disease^31^,^32^,^33^; further excluded was one publication^34^ which was an overlapping report of another^35^ with larger events. Finally, 10 publications^13^,^14^,^15^,^16^,^17^,^18^,^19^,^20^,^21^,^22^ including 11 independent prospective studies (2 cohorts were combined in one publication^17^) were included in this meta-analysis, involving 7 studies^13^,^14^,^15^,^16^,^17^,^18^ on dietary n-3 LCPUFA and 4 studies^19^,^20^,^21^,^22^ on circulating EPA/DHA in relation to all-cause mortality risk. These studies covered a total of 371 965 participants from general populations and 31 185 death events.

The 7 studies on dietary n-3 LCPUFA were published between 2004 and 2015, covering 361 273 participants and 27 624 deaths during 5.0 to 24 years of follow-up. Dietary intakes were mostly assessed with self-administered food frequency questionnaires (FFQ), with the exception of one Japanese cohort^15^ in which food records were applied. N-3 LCPUFA consisted of both EPA and DHA in all studies and the intakes were estimated from food sources in 6 studies^14^,^15^,^16^,^17^,^18^ and from both food and supplementation in one^13^. In all four studies that were published between 2008 and 2015, circulating n-3 LCPUFA were measured by gas chromatography. A total of 3561 death events were identified from 10 692 participants during 9.6 to 30.7 years of follow-up. All of the 4 studies separately reported results for EPA and DHA. The characteristics of the included studies are summarized in Table 1. All of the included studies provided risk estimates that were controlled for multi-variables.

### N-3 LCPUFA intake and all-cause mortality

**Figure 2:**
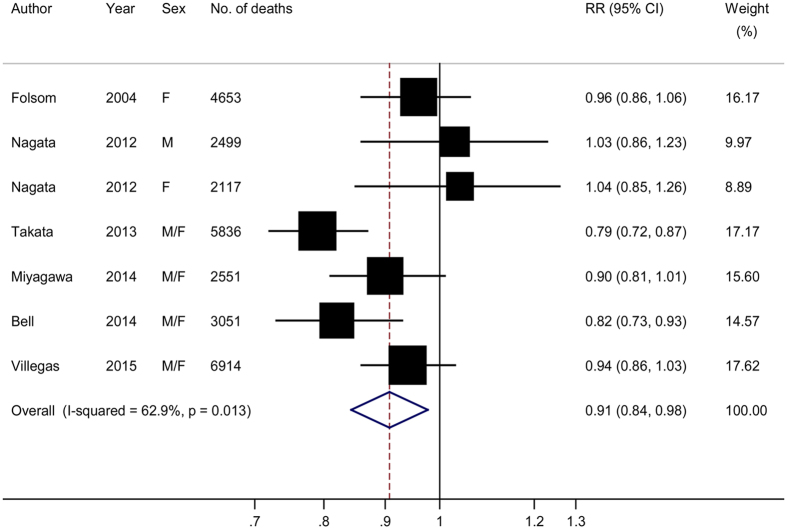
Risk estimates of all-cause mortality for the highest compared with lowest intake of long-chain n-3 polyunsaturated fatty acids in individual studies and all combined. F, female; M, male.

A meta-analysis of the 7 prospective studies suggested a summary RR of 0.91 (95% CI: 0.84–0.98) for the highest compared with lowest categories of n-3 LCPUFA intake, with moderate heterogeneity (*P*-heterogeneity = 0.01, *I*^2^ = 62.9%) (Fig. 2). There was no evidence of publication bias (*P* values for Egger and Begg tests ≥0.30). Meta-regression analysis showed that the observed heterogeneity was not explained by pre-defined study and population characteristics (Table 2, *P* values for difference ≥0.14). A sensitivity analysis conducted by omitting one study at each turn showed a RR range of 0.88 (95% CI: 0.81–0.95) to 0.93 (95% CI: 0.87–0.99), and the overall *I*^2^ reduced from 62.9% to 32.3% when the Chinese cohorts by Takata *et al*.^17^ were omitted. Three independent studies (2 publications^13^,^17^) reported separate results for EPA and DHA intake in relation to all-cause mortality, and the summary high-versus-low RR was 0.83 (95% CI: 0.75–0.92, *I*^2^ = 51.5%) for EPA and 0.81 (95% CI: 0.74–0.95, *I*^2^ = 38.5%) for DHA, respectively.

### Circulating EPA and DHA and all-cause mortality

**Figure 3:**
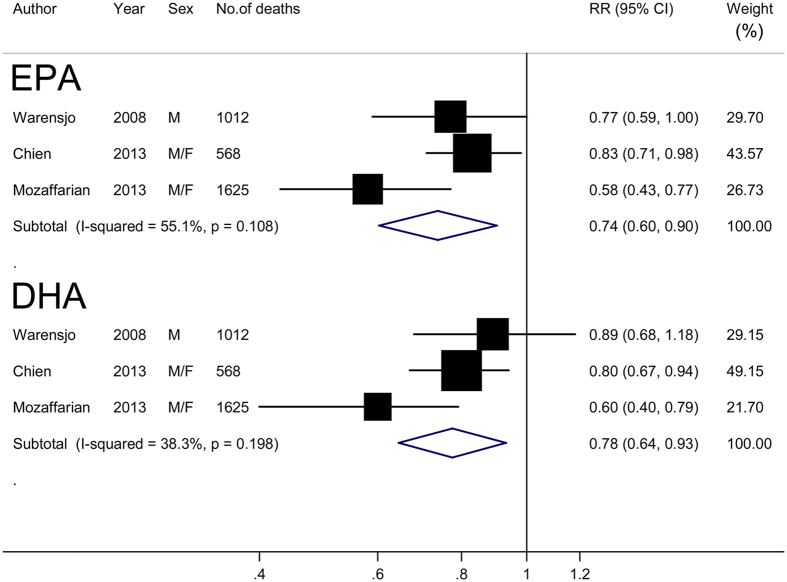
Risk estimates of all-cause mortality for the highest compared with lowest proportions of circulating eicosapentaenoic acid (EPA) and docosahexaenoic acid (DHA) to total fatty acids in blood for individual studies and all combined. F, female; M, male.

Three of the 4 studies reported high-versus-low circulating EPA and DHA and risk of all-cause mortality. The summary RR was 0.74 (95% CI: 0.60–0.90) for EPA and 0.78 (95% CI: 0.64–0.93) for DHA, with moderate heterogeneity (*I*^2^ = 55.1% and 38.3%, respectively) (Fig. 3). There was no evidence of publication bias (all *P* values ≥ 0.30).

### Dose-response analysis

**Figure 4:**
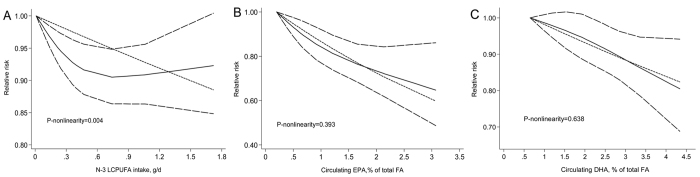
Risk estimates of all-cause mortality associated with dietary long-chain n-3 polyunsaturated fatty acids (panel A) and circulating eicosapentaenoic acid (panel B) and docosahexaenoic acid (panel C) in a restricted cubic spline random-effects meta-analysis. FA, fatty acids; LCPUFA, long-chain polyunsaturated fatty acids.

One study^16^ on dietary n-3 LCPUFA intake was not included in these analyses because the levels of the intake for each category were not available. Pooling the remaining 6 studies showed a summary RR of 0.94 (95% CI: 0.89–0.99) for an increment in n-3 LCPUFA intake of 0.3 g/d, with moderate heterogeneity (*P*-heterogeneity = 0.01, *I*^2^ = 70.2%) (Supplementary Fig. S1). There was evidence of a nonlinear association (*P*-nonlinearity = 0.004) (Fig. 4A), with a tendency to plateau at high intakes (>0.6 g/d). However, this observation should be treated with caution because all data for high intakes were from one Japanese cohort^15^.

All four studies were included in the dose-response analysis of circulating EPA and DHA and all-cause mortality. The summary RR was 0.80 (95% CI: 0.65–0.98) and 0.79 (95% CI: 0.63–0.99) for each 1% increment in the proportions of EPA and DHA in total circulating fatty acids, with moderate to high heterogeneity (*I*^2^ = 74.5% and 79.3%, respectively) (Supplementary Fig. S2). There was no evidence of a nonlinear assocation between circulating EPA or DHA and all-cause mortality (*P* values for nonlinearity >0.30) (Fig. 4B,C). There was no evidence of publication bias across the dose-response analyses (all *P* values ≥ 0.09).

## Discussion

### Major findings

In this meta-analysis involving over 30 thousand deaths events from 11 prospective studies, both dietary and circulating n-3 LCPUFA are shown to be significantly associated with reduced risk of all-cause mortality, and the associations are similar for EPA and DHA.

### Other results from observational studies and clinical trials

In a recent meta-analysis of 12 prospective studies, Zhao *et al*.^36^ report a moderate reduction in all-cause mortality associated with intake of fish, a major dietary source of n-3 LCPUFA. Significantly inverse associations of dietary fish and dietary and circulating n-3 LCPUFA with risk of CHD have been consistently shown in prospective observational studies^2^,^10^. Conversely, clinical evidence regarding the health benefits of n-3 LCPUFA has been continuously inconsistent. Mozaffarian and Wu^5^ review the cumulative observational and clinical evidence published before 2011 regarding the effects of n-3 LCPUFA on CVD and related risk factors, and conclude that there is strong evidence supporting a protective effect of n-3 LCPUFA on cardiac death. Nonetheless, more recent meta-analyses fail to show any significant protection either on CVD or on all-cause mortality. For instance, Rizos *et al*.^11^ and Chowdhury *et al*.^10^ each pooled data from 17 RCTs and report no significant effect of n-3 LCPUFA supplementation on all-cause mortality, with a summary RR of 0.96 (95% CI, 0.91–1.02) and 0.94 (95% CI: 0.86–1.03), respectively. Using a cumulative meta-analysis, Rizos *et al*.^11^ further find that a protection of n-3 LCPUFA on all-cause mortality is restricted to RCTs that are published before 2007.

The reasons for the disparate findings between observational and clinical studies are not fully understood, but several possibilities merit consideration. First, most of the RCTs, as argued by Mozaffarian and Wu^5^, are small in sample size and short in duration, and therefore may be of a low statistical power to detect a moderate-to-weak effect associated with long-term uses. Second, participants in the trials are mostly those subjects who are at high risk for, or already suffering from CVD rather than the general populations. In such a condition, the benefits of n-3 LCPUFA may be diminished or offset by disease status or corresponding dietary modifications and medical treatments. A recent review^37^ summarize that post-hoc analyses of RCTs support beneficial effects of n-3 LCPUFA on CVD prevention among statins non-users, and conclude that emerging uses of statins and less deficiencies of n-3 LCPUFA among participants of recent trials may explain why especially early RCTs, but not recent ones find the health benefits of n-3 LCPUFA. Given that n-3 LCPUFA and statins share several mechanisms whereby they may exert health effects (e.g., improving endothelial function, reducing inflammation, and slowing atherosclerotic progresses^5^,^38^), statins may mask the action of n-3 LCPUFA in a competitive fashion.

Third, the association of n-3 LCPUFA with health outcomes may be nonlinear. A 2006 pooled analysis^39^ of prospective studies and randomized trials indicate that the most protections of EPA and DHA intake on CHD deaths could be achieved when the intake is up to 0.25 g/d. Furthermore, the effects on antiarrhythmia, antithrombosis, and reducing heart rate and blood pressure (but not triglycerides) also appear nonlinear, with tendencies to plateau at the intakes of 0.5–0.75 g/d^39^. A recent report from the Cardiovascular Health Study^21^ demonstrate that EPA and DHA in plasma increase linearly and sharply with increasing dietary EPA and DHA lower than 0.5 g/d, whereas there are limited subsequent change in plasma concentrations despite larger increases in dietary intake. These observations suggest that diet and endogenous metabolism may jointly determine to what degree n-3 LCPUFA intake may exert their benefits to human body. If the benefits of n-3 LCPUFA indeed tend to be saturable at low-to-moderate levels, the null effects observed in the trials without taking into account diet background of participants are not surprising.

Fourth, it is possible that other nutrients (e.g., vitamins, minerals, and proteins) in fish or other foods rather than n-3 LCPUFA are beneficial. It is difficult, perhaps not possible for observational studies to accurately distinguish the effects between n-3 LCPUFA and these nutrients. Finally, even if n-3 LCPUFA are one of the causal components in fish, their consumption as part of a matrix of other nutrients in foods may be essential for the benefits. We consider the fourth possibility less likely because of multiple lines of evidence from experimental research, prospective observational studies, as well as human intervention trials supporting the potential cardiovascular benefits of n-3 LCPUFA.

### Strengths and limitations of the current study

Major strengths of this meta-analysis include the prospective design of original studies and the large number of events involved in the analyses. However, several limitations of this study should also be acknowledged. Since this meta-analysis is based on observational studies, potential influences of residual or unmeasured confounders on our findings cannot be fully excluded. Dietary information was mostly collected with self-reported FFQs in the original studies, which may introduce measurement error and lead some participants to be misclassified. Such misclassification would likely be nondifferential in cohort studies and to attenuate any true association. This may partly explain the observation that the associations between circulating n-3 LCPUFA and all-cause mortality were stronger than those between dietary intakes and all-cause mortality. Furthermore, the common methods we used to detect potential publication bias may be of a limited power when the number of studies is relatively small. Thus, potential impacts of publication bias on our results cannot be completely excluded.

### Conclusions

In summary, this meta-analysis of prospective observational studies suggests that both dietary and circulating n-3 LCPUFA are significantly inversely associated with risk of all-cause mortality. More large prospective studies conducted among individuals with high intakes are needed to address whether there is a nonlinear association. Future well designed primary prevention trials that account for nutrition status, health conditions, and medication usages of participants are also warranted to confirm our findings and those from others.

## Methods

### Literature search

This study was planned, conducted, and reported in adherence to the guidelines of the ‘Meta-analysis Of Observational Studies in Epidemiology (MOOSE) group’^40^. A literature search was performed on PubMed (January 1, 1966 to November 30, 2015) and EMBASE (January 1, 1980 to November 30, 2015) databases using the search strategy as follows: (n-3 fatty acids OR omega-3 fatty acids OR marine fatty acids OR n-3 polyunsaturated fatty acids OR n-3 PUFA OR docosahexaenoic acid OR eicosapentaenoic acid OR DHA OR EPA) AND (mortality OR death) AND (cohort OR prospective OR nested). Bibliographies in the retrieved full articles were also carefully hand searched for additional studies. Attempts were also made to contact relevant authors for additional information.

### Study selection

Studies that met the following criteria were considered: *1)* the study design was prospective; *2)* the exposure of interest was dietary or circulating n-3 LCPUFA; *3)* the outcome of interest was all-cause mortality *4)* relative risks (RRs) with corresponding 95% confidence intervals (CIs) were reported or could be estimated. When multiple publications from the same study were available, the one with the largest number of events was selected.

### Data extraction and quality assessment

Using a standardized data-collection form, the following data were extracted from each included study: the first author’s last name, publication year, country of origin, source of populations, study duration, age and sex of participants, number of events and participants, categories of n-3 LCPUFA, the maximally adjusted RRs with 95% CIs, methods for exposure assessments, and potential confounders accounted for in the statistical model. The study quality was evaluated with the 9-star Newcastle-Ottawa Scale (NOS)^41^. Literature selection, data extraction and quality assessment were conducted independently by two authors (G-CC and L-QQ), with any disagreement resolved by consensus.

### Statistical analysis

In a Japanese cohort^15^, mortality risks associated with n-3 LCPUFA intake were reported by causes of deaths (CVD and non-CVD). We combined results from the sub-cohorts with a fix-effects model, and included the overall estimates in the meta-analysis. A DerSimonian and Laird random-effects model^42^, which considers both within-and between-study variation was assigned to calculate the summary risk estimates. Heterogeneity test was performed using *Q* and *I*^2^ statistics^43^. For the *Q* statistic, *P* < 0.1 was considered as statistically significant; and for the *I*^2^ statistic, the following cut-off points were used: <30% (little or no heterogeneity), 30–75% (moderate heterogeneity) and >75% (high heterogeneity). Potential publication bias was investigated with both Egger regression and Begg correlation tests^44^,^45^. Meta-regression analyses were performed to explore potential sources of heterogeneity according to geographic area, duration of follow-up, sex and age of participants, range of the exposure, and quality score of included studies. We also separately evaluated mortality risks associated with dietary/circulating EPA and DHA.

Given the distinct cut-off points across studies, dose-response analyses were performed with the method proposed by Greenland and Longnecker^46^ and Orsini *et al*.^47^. The method requires the number of cases and person-years and the risk estimates with their variance for at least 3 quantitative exposure categories. For studies that did not provide the number of cases/person-years in each exposure category, the data were estimated from total number of cases/person-years. For each study, the median/mean level of exposure for each category was assigned to each corresponding risk estimate. When the median/mean level per category was not provided, the midpoint of the upper and lower boundaries in each category was assigned as an average level. If the highest category was open-ended, the width of the interval was assumed to be the same as in the second highest category. For 1 study^19^ where the categorized levels of circulating EPA and DHA were not provided, we contacted the corresponding author and obtained the data. For 2 studies^20^,^22^ where results for circulating EPA and DHA were reported as a continuous variable (1-SD increase), we rescaled the RR to a 1% increase in circulating EPA and DHA. One of the 2 studies did not report SD values, and the values were estimated using reported inter-quartile ranges according to the methods developed by Hozo *et al*.^48^. The results of linear dose-response analyses were presented for a 0.3 g/day increment in dietary n-3 LCPUFA (approximates 1 serving/week of fatty fish intake), and for a 1% increment in circulating EPA/DHA. We further examined a potential nonlinear relationship between dietary/circulating n-3 LCPUFA and all-cause mortality by modeling exposure levels using restricted cubic splines with 3 knots at percentiles 10%, 50% and 95% of the distribution^49^,^50^. A *P* value for nonlinearity was calculated by testing the null hypothesis that the coefficient of the second spline is equal to zero. All statistical analyses were performed using STATA software, version 11.0 (STATA Corp., College Station, TX, USA).

## Additional Information

**How to cite this article**: Chen, G.-C. *et al*. N-3 long-chain polyunsaturated fatty acids and risk of all-cause mortality among general populations: a meta-analysis. *Sci. Rep.* **6**, 28165; doi: 10.1038/srep28165 (2016).

## Supplementary Material

Supplementary Information

## Figures and Tables

**Table 1:** Prospective studies that investigated the association of dietary and circulating EPA and/or DHA with risk of all-cause mortality.

First author, year (Country/region)	Source of populations, duration	Participants	No. of deaths	Comparison	RR (95% CI)	Diet assessment	Adjustment for potential confounders	Quality scores
***Dietary intake***								
Folsom, 2004 (United States)	IWHS, 14yr	41 836 F aged 55–69 yr	4653	Q5 vs. Q1	0.96 (0.86–1.06)	Self-reported FFQ	Age, BMI, WHR, education, physical activity, smoking, age at first live birth, estrogen use, vitamin use, diabetes, hypertension, and intake of energy, alcohol, whole grains, fruit and vegetables, red meat, cholesterol, and saturated fat.	7
Nagata, 2012 (Japan)	Takayama Study, 16 yr	28 356 M/F aged ≥35 yr	2499 M; 2117 F	Q5 vs. Q1	1.03 (0.86–1.23) (M) 1.04 (0.85–1.26) (F)	Self-reported FFQ, validated	Age, height, BMI, physical activity, smoking, education, marital status, histories of diabetes and hypertension, and intakes of energy, alcohol, protein, SFA, MUFA, non-long-chain n-3 PUFA, fruit, vegetables, fiber, and percent energy from carbohydrate in foods other than rice.	9
Takata, 2013 (China)	SMHS, 5.6 yr; SWHS, 11.2 yr	61 137 M aged 40–74 yr; 73 159 F aged 40–70 yr	2170 M; 3666 F	Q5 vs. Q1	0.79 (0.72–0.87)	Self-reported FFQ, validated	Age, income, occupation, education, comorbidity index, physical activity, smoking, and intakes of energy, alcohol (for M), red meat, poultry, fruit, and vegetable.	8
Miyagawa, 2014 (Japan)	NIPPON DATA80, 24 yr	9190 M/F aged ≥30 yr	2551	Q5 vs. Q1	0.80 (0.66–0.96)^a^ 0.97 (0.84–1.12)^b^	Food records	Age, sex, BMI, smoking, SBP, blood glucose, serum total cholesterol, antihypertensive medication status, residential area, intakes of alcohol SFA, n-6 PUFA, vegetable protein, total dietary fiber, and sodium.	8
Bell, 2014 (United States)	VITAL Study, 5.0 yr	70 495 M/F aged 50–76 yr	3051	Q4 vs. Q1	0.84 (0.76–0.93)	Self-reported FFQ	Age, sex, BMI, smoking, race/ethnicity, marital status, education, physical activity, self-rated health, mammogram in, prostate- specific antigen test, sigmoidoscopy, uses of cholesterol-lowering medication, aspirin, non-aspirin NSAIDs, estrogen, and estrogen+progestin, morbidity score, age at menopause, age at death of father or mother, and intakes of total energy and energy from trans fat and SFA, alcohol, fruit, and vegetables,	7
Villegas, 2015 (United States)	SCCS, 5.5 yr	77 100 M/F aged 40–79 yr	6917	Q5 vs. Q1	0.94 (0.86–1.03)	Self-reported FFQ, validated	Age, sex, BMI, smoking, physical activity, income, education, insurance coverage, race, and intakes of energy, alcohol, and total meat.	8
***Circulating levels***								
Warensjö, 2008 (Sweden)	ULSAM, 30.7yr	1885 M aged 50 yr	1012	Per SD^c^ increase	EPA: 1.00 (0.94–1.08) DHA: 0.95 (0.89–1.02)	Gas chromatography	Total cholesterol, BMI, smoking, physical activity, and hypertension.	8
Chien, 2013 (Taiwan)	Residents living in Chin-Shan Township, 9.6 yr	1833 M/F aged >35 yr	568	Q4 vs. Q1	EPA: 0.77 (0.59–1.00) DHA: 0.89 (0.68–1.18)	Gas chromatography	Age, sex, BMI, smoking, alcohol drinking, marital status, education, occupation, sports activity, hypertension, diabetes, LDL-C and HDL-C levels.	8
Mozaffarian, 2013 (United States)	CHS	2692 M/F aged ≥65 yr	1625	Q5 vs. Q1	EPA: 0.83 (0.71–0.98) DHA: 0.80 (0.67–0.94)	Gas chromatography	Age, sex, BMI, WC, physical activity, race, education, enrollment site, fatty acids measurement batch, smoking, prevalent diabetes, AF, and drug- treated hypertension, and intakes of alcohol, tuna or other broiled or baked fish, fried fish, red meat, fruit, vegetables, and dietary fiber.	7
Marklund, 2015 (Sweden)	Residents living in Stockholm County, 14.5 yr	4232M/F aged 60 yr	356	Q4 vs. Q1^d^	EPA: 0.81 (0.72–0.91) DHA: 0.75 (0.68–0.84)	Gas chromatography	Sex, BMI, smoking, physical activity, education, alcohol intake, diabetes mellitus, drug-treated hypertension, and drug-treated hypercholesterolemia.	8

AF, atrial fibrillation; BMI, body mass index; CHS, Cardiovascular Health Study; CVD, cardiovascular disease; DHA, docosahexaenoic acid; EPA, eicosapentaenoic acid; F, females; FFQ, Food frequency questionnaire; HDL-C, high-density lipoprotein cholesterol; IWHS, Iowa Women’s Health Study; LDL-C, low-density lipoprotein cholesterol; M, males; MUFA, monounsaturated fatty acid; NSAIDs, non-aspirin nonsteroidal anti-inflammatory drugs; PUFA, polyunsaturated fatty acid; Q, quartile/quintile; SCCS, Southern Community Cohort Study; SBP, systolic blood pressure; SFA, saturated fatty acids; SMHS, Shanghai Men’s Health Study; SWHS, Shanghai Women’s Health Study; ULSAM, The Uppsala Longitudinal Study of Adult Men; WC, waist circumference; WHR, waist/hip ratio; yr, years.

^a^Risk estimates for total CVD mortality.

^b^Risk estimates for total non-CVD mortality.

^c^The estimated SD was 0.52% for EPA and 0.19% for DHA, respectively.

^d^This study also reported risk estimates for per SD (1% for EPA and 0.2% for DHA, respectively) increase in circulating long-chain n-3 fatty acids, and presented sex-specific results.

**Table 2:** Subgroup analysis for the association of n-3 LCPUFA intake (high vs. low) and risk of all-cause mortality.

	*N*	RR (95% CI)	*P*_heterogeneity_	*I*^2^(%)	*P*_difference_
Overall	7	0.90 (0.83–0.98)	0.005	70.0	
Area					
Asian	4	0.90 (0.77–1.05)	0.003	82.6	0.91
USA	3	0.91 (0.83–0.99)	0.12	53.4	
Duration					
≥10 years	5	0.91 (0.81–1.03)	0.004	77.8	0.74
<10 years	2	0.88 (0.77–1.01)	0.08	68.2	
Sex					
Male	1	1.03 (0.86–1.23)	–	–	Ref.
Female	2	0.98 (0.90–1.07)	0.45	0	0.72
Both	5	0.86 (0.79–0.94)	0.05	62.7	0.24
Mean/median age at baseline					
≥55 years	2	0.89 (0.76–1.04)	0.05	73.2	0.75
<55 years	5	0.92 (0.83–1.01)	0.01	67.8	
Range of intake^a^					
≥0.30 g/d	4	0.91 (0.85–0.97)	0.22	31.4	0.14
<0.30 g/d	2	0.79 (0.72–0.87)	–b	–b	
Quality scores					
≥8	5	0.91 (0.82–1.01)	0.005	76.8	0.84
<8	2	0.89 (0.76–1.04)	0.05	73.2	
Subtypes					
EPA	3	0.83 (0.75–0.92)	0.15	51.5	0.83
DPA	3	0.81 (0.74–0.90)	0.20	38.5	

DHA, docosahexaenoic acid; EPA, eicosapentaenoic acid; LCPUFA, long-chain polyunsaturated fatty acids.

^a^The mean/median intakes in the highest categories minus those in the lowest categories was the range of intake. This analysis excluded the study by Nagata *et al*. in which the intake levels for each category were not reported.

^b^Only two cohorts from one publication were included in this stratum, and so no result for heterogeneity test were reported here.
