# Supplementary material for: N-3 long-chain polyunsaturated fatty acids and risk of all-cause mortality among general populations: a meta-analysis
Source: Sci Rep. 2016 Jun 16;6:28165. doi: 10.1038/srep28165 (PMC4910132; doi:10.1038/srep28165)
Supplement: Supplementary Information [file srep28165-s1.pdf]

N-3 long-chain polyunsaturated fatty acids and risk of all-cause mortality among general populations: a meta-analysis

Guo-Chong Chen[1], Jing Yang[1,2], Manfred Eggersdorfer[3], Weiguo Zhang[4*], and Li-Qiang Qin[1*]
[1]Department of Nutrition and Food Hygiene, School of Public Health, Soochow University, Suzhou, China.
[2]Department of Nutrition, the First Affiliated Hospital of Soochow University, Suzhou, China
[3]DSM Nutrition Products, Human Nutrition and Health, Kaiseraugst, Switzerland
[4]DSM Nutrition Products, Human Nutrition and Health, Beijing, China

Supplementary figure captions:

Supplementary Figure S1. Risk estimtes of all-cause mortality for an increase in long-chain n-3 fatty acids intake of 0.3 g/d in individual studies and all combined. F, female; M, male.

Supplementary Figure S2. Risk estimtes of all-cause mortality for each 1% increment in the proportions of circulating eicosapentaenoic acid (EPA) and docosahexaenoic acid (DHA) to total fatty acids in blood for individual studies and all combined. F, female; M, male.

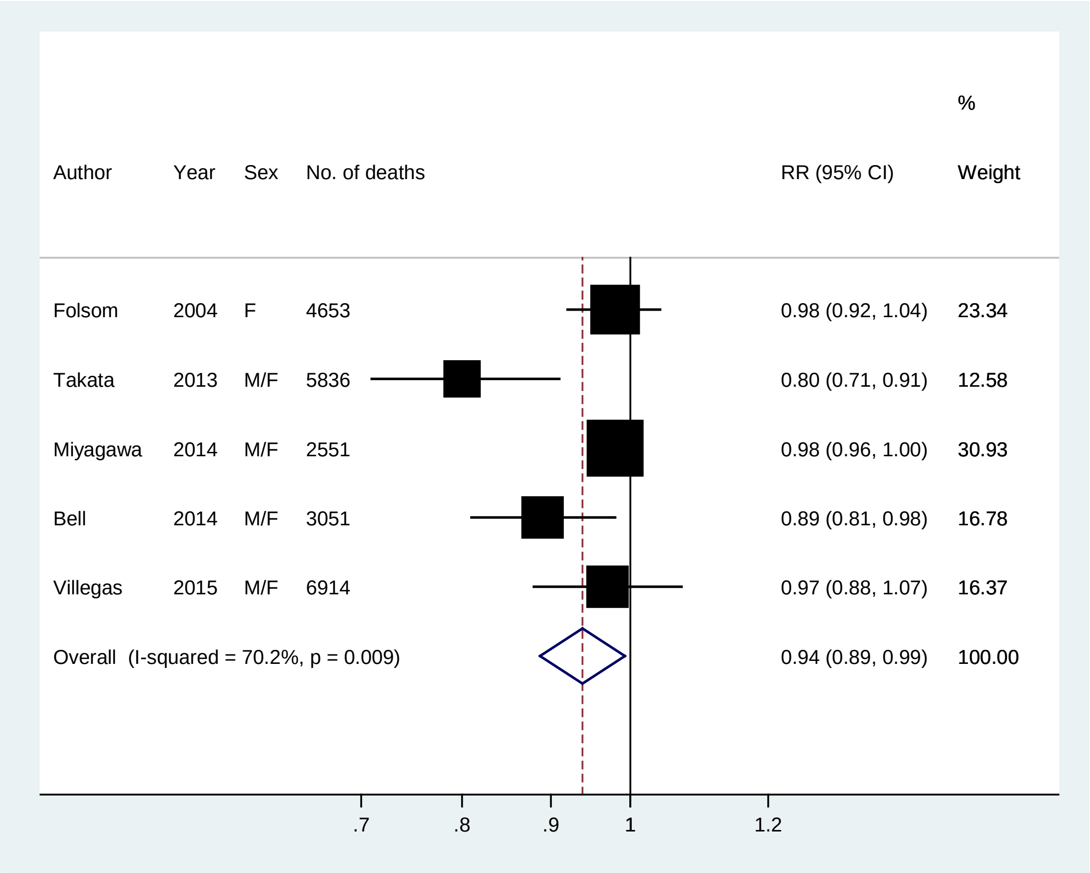

Supplementary Figure S1: Risk estimtes of all-cause mortality for an increase in long-chain n-3 fatty acids intake of 0.3 g/d in individual studies and all combined. F, female; M, male.

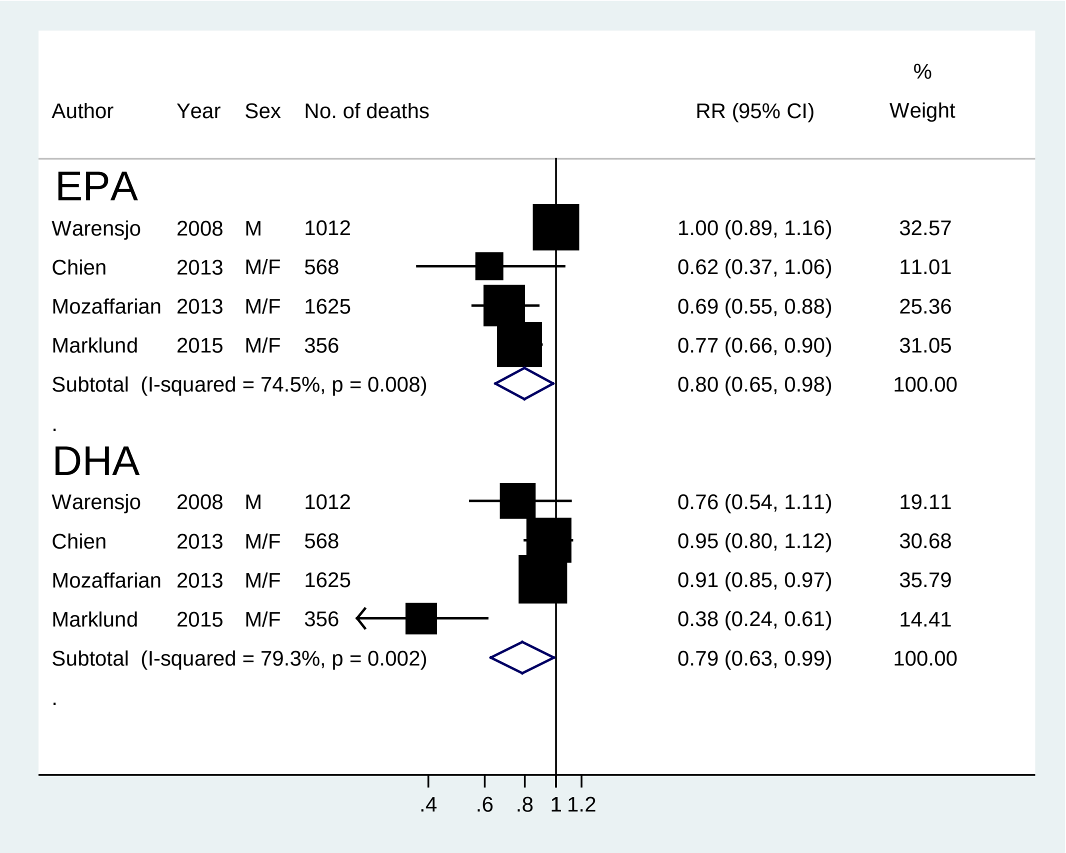

Supplementary Figure S2: Risk estimtes of all-cause mortality for each 1% increment in the proportions of circulating eicosapentaenoic acid (EPA) and docosahexaenoic acid (DHA) to total fatty acids in blood for individual studies and all combined. F, female; M, male.
